# Letter from the Editor in Chief

**DOI:** 10.19102/icrm.2024.15106

**Published:** 2024-10-15

**Authors:** Devi Nair


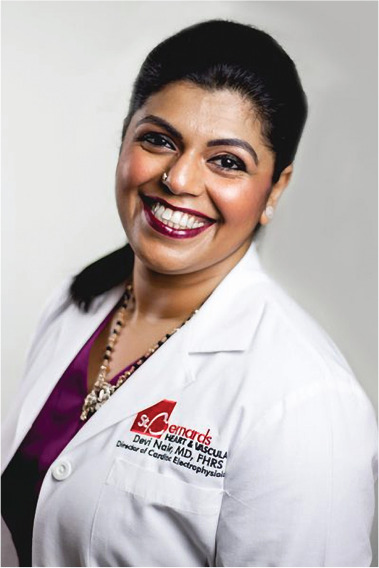


Dear Readers,

I am delighted to present this special October issue of *The Journal of Innovations in Cardiac Rhythm Management*, which brings you highlights from the latest cutting-edge research unveiled at the Asia Pacific Heart Rhythm Society (APHRS) 2024 conference in Sydney. Attending APHRS 2024 allowed me to experience firsthand the unveiling of pivotal late-breaking trials that underscore the dynamism and promise in our field. I am excited to share some of these landmark findings with you, as they hold the potential to redefine current standards and advance patient care in cardiac rhythm management.

The International Collaborative Left Bundle Branch Area Pacing Study (I-CLAS) generated significant interest at APHRS 2024. This large, multi-center study evaluated the efficacy of conduction system pacing (CSP), particularly left bundle branch area pacing, compared to traditional biventricular pacing (BVP) for cardiac resynchronization therapy (CRT) in heart failure patients with mildly reduced ejection fraction and left bundle branch block (LBBB). The findings were remarkable: CSP led to a significant reduction in the combined endpoint of heart failure hospitalizations and all-cause mortality when compared to BVP, especially in patients with LBBB. This shift toward CSP, offering more physiological pacing, signals a possible new standard in CRT approaches, with CSP potentially becoming the preferred CRT strategy in patients who meet these criteria.

Another landmark study presented was the Extravascular ICD Pivotal Study, which assessed the long-term performance and safety of the extravascular implantable cardioverter-defibrillator (EV-ICD). With a substernal lead position, the EV-ICD aims to provide the benefits of a traditional transvenous ICD without the associated vascular complications. Over a mean follow-up of 30.6 months, the EV-ICD demonstrated a 100% success rate in terminating ventricular arrhythmias and a 77% success rate for anti-tachycardia pacing. Furthermore, the complication rate remained low at 11%, with no unique complications, such as sepsis or mediastinitis, observed. This innovation has implications for high-risk patients needing ICD therapy, as it provides an effective, less-invasive option that maintains reliable arrhythmia control.

The application of pulsed field ablation (PFA) was also a focal point, particularly in the Pulmonary Vein Isolation Durability with a Conformable Single-Shot Pulsed Field Ablation Catheter study, led by Dr. Vivek Reddy and colleagues. This study showcased a conformable PFA catheter capable of achieving durable pulmonary vein isolation (PVI) with fewer applications per vein. Data indicated that acute PVI was achieved successfully in all treated patients while maintaining a low complication profile, underscoring the safety and potential of PFA for routine atrial fibrillation (AF) management. Notably, the study also included optional invasive remapping, which revealed sustained PVI at 75 days in most patients, supporting PFA as a highly effective approach for paroxysmal AF with durable outcomes.

In addition to these advancements, I was honored to present my own research assessing the durability of PVI achieved with the PulseSelect catheter (Medtronic, Minneapolis, MN, USA). This study employed invasive remapping to verify lesion durability post-ablation, yielding promising insights into the catheter’s effectiveness in maintaining PVI over time. Results demonstrated that a high proportion of treated veins remained isolated on remapping, indicating robust lesion durability and supporting the PulseSelect catheter as a reliable option for achieving and maintaining PVI in patients with atrial fibrillation. The use of invasive mapping provided a granular view of ablation efficacy, which could inform future best practices for achieving optimal outcomes with PFA.

A study from the Chinese University of Hong Kong assessed the long-term effects of PFA on coronary arteries. Despite measures to mitigate acute coronary spasm during atrial flutter ablation, a modest increase in vascular wall area and a 10% reduction in luminal area were observed at a 3-month follow-up. Although the risk of coronary stenosis appears manageable, these findings underscore the importance of long-term follow-up and vigilance in patients undergoing PFA for atrial arrhythmias.

Finally, the new Aveir AR leadless atrial pacemaker (Abbott, Chicago, IL, USA), which recently gained commercial release, was evaluated for its safety and efficacy in patients with sinus node dysfunction. In a cohort of 42 patients, the Aveir AR pacemaker achieved a 100% implant success rate, with no complications observed within the 30-day follow-up. This innovative pacemaker demonstrated stable electrical metrics and represents an important step forward in leadless pacemaker technology and physiologic pacing, offering a minimally invasive solution specifically for atrial pacing needs.

The APHRS 2024 conference showcased the forefront of research, underscoring the field’s rapid advancement toward personalized, minimally invasive therapies. This issue of *The Journal of Innovations in Cardiac Rhythm Management* encapsulates these developments, highlighting trials and technologies poised to elevate standards of care and improve patient outcomes in cardiac rhythm management. I extend my gratitude to the researchers, clinicians, and the APHRS team led by Dr. Prashanthan Sanders for their contributions to this year’s conference and their dedication to advancing electrophysiology.

Thank you for joining us in exploring these breakthrough studies. I look forward to witnessing how these findings will shape the future of our field and am eager to continue sharing such transformative research with our readers in the coming months.

Warm regards,


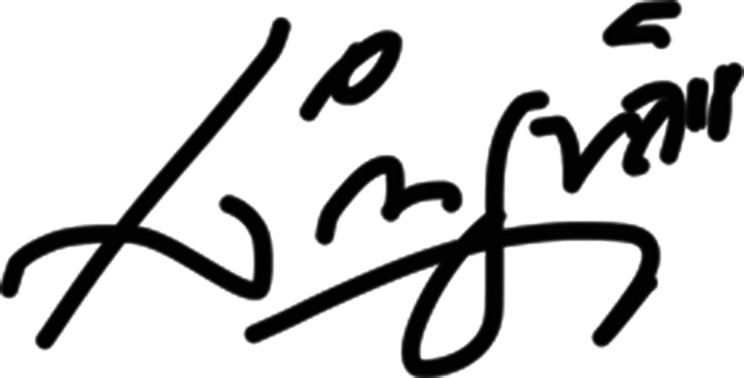


Dr. Devi Nair, md, facc, fhrs

Editor-in-Chief

*The Journal of Innovations in Cardiac Rhythm Management*

Director of the Cardiac Electrophysiology & Research,

St. Bernard’s Heart & Vascular Center, Jonesboro, AR, USA

White River Medical Center, Batesville, AR, USA

President/CEO, Arrhythmia Research Group

Clinical Adjunct Professor, University of Arkansas for Medical Sciences

Governor, Arkansas Chapter of American College of Cardiology

drdgnair@gmail.com

